# Application of the Enhanced Recovery After Surgery (ERAS) programme in elective colorectal resection for diverticular disease: a retrospective propensity score-matched cohort study

**DOI:** 10.1007/s00384-026-05121-x

**Published:** 2026-03-16

**Authors:** Diletta Cassini, Sara Lauricella, Francesco Brucchi, Francesca De Stefano, Stefano Clementi, Giuseppe Faillace, Gianandrea Baldazzi

**Affiliations:** 1 Unit of General Surgery, General and Emergency Unit, Sesto San Giovanni Hospital, ASST Nord Milano, 20099 Sesto San Giovanni, Italy; 2 Colorectal Surgery Unit, Fondazione IRCCS Istituto Nazionale Dei Tumori, 20133 Milan, Italy; 3 General Surgery Residency Program, University of Milan, 20122 Milan, Italy; 4 Department of Anaesthesia, Sesto San Giovanni Hospital, Milan, Italy; 5 Department of General and Emergency Surgery, Legnano Hospital, Legnano, Italy

**Keywords:** Enhanced Recovery after Surgery (ERAS), Diverticular Disease, Acute Diverticulitis, Elective Sigmoid Resection, Benign Colorectal Surgery, Postoperative Outcomes, Length of Stay (LOS), Minimally Invasive Colorectal Surgery, Perioperative Care Pathways, Surgical Recovery

## Abstract

**Background:**

Diverticular disease is one of the most common benign colorectal conditions and often requires elective resection for recurrent or complicated presentations. Enhanced Recovery After Surgery (ERAS) programmes have demonstrated benefits in mixed colorectal populations; however, evidence in purely benign diverticular cohorts remains limited. This study evaluated the impact of a standardised ERAS pathway on postoperative outcomes following elective colorectal resection for diverticular disease.

**Methods:**

This retrospective multicentre cohort study included consecutive adults undergoing elective left-sided colorectal resection for diverticular disease between 2009 and 2024 in Northern Italy. Patients treated within an ERAS pathway were compared with those receiving conventional care. The primary outcome was length of hospital stay. Secondary outcomes included postoperative morbidity, gastrointestinal recovery, pain, mobilisation, and readmissions. Continuous variables were analysed with the Mann–Whitney U test and categorical variables with χ^2^ or Fisher’s exact test. A propensity score–matched analysis was performed to account for baseline and temporal confounding.

**Results:**

A total of 421 patients were included: 329 in the ERAS group and 92 in the non-ERAS group. Baseline characteristics were similar. ERAS adherence was associated with faster gastrointestinal recovery (median time to first flatus: 1 vs 2 days; stool: 1 vs 2 days), lower pain on POD 1 (VAS 2 vs 4), earlier mobilisation (12 h vs 21 h), and earlier solid diet introduction (POD 1 vs POD 2). Overall morbidity was lower in the ERAS group (6.6% vs 14%), without increases in severe complications or readmissions. Median LOS was reduced (4 vs 6 days). Propensity score matching (88 pairs) confirmed these findings.

**Conclusion:**

ERAS implementation in elective colorectal resection for diverticular disease is safe, feasible, and associated with accelerated recovery, reduced morbidity, and shorter hospital stay.

## Introduction

Diverticular disease is one of the most common benign colorectal conditions in Western countries, with a rising prevalence and a substantial impact on healthcare systems [1, 2]. Elective sigmoid resection is typically offered to patients with recurrent or persistent symptoms following episodes of acute diverticulitis, or in the presence of complications such as fistula, stenosis, or recurrent inflammatory masses. Contemporary guidelines emphasise an individualised, symptom-driven approach to surgical indication—prioritising the impact on quality of life—rather than adherence to a fixed threshold based solely on the number of previous attacks [3, 4]. Additional evidence supports the applicability of Enhanced Recovery After Surgery (ERAS) programmes in diverticulitis. A large retrospective cohort study including 1,730 patients undergoing elective colectomy within ERAS pathways reported no significant differences in key postoperative outcomes between those treated for diverticulitis and those undergoing surgery for colorectal neoplasia [5]. Rates of prolonged length of stay (LOS), surgical site infection, serious morbidity or mortality, reoperation, and readmission were comparable across diagnoses, suggesting that ERAS protocols confer similar benefits in benign inflammatory conditions as in malignant disease.

Despite advances in minimally invasive surgery, elective resection for diverticular disease remains associated with relevant postoperative morbidity, prolonged LOS, and a non-negligible risk of readmission, particularly in older or frail patients with multiple comorbidities [6]. These considerations highlight the importance of structured perioperative pathways that optimize physiological recovery. Enhanced Recovery After Surgery (ERAS) programmes achieve this through coordinated, evidence-based interventions-including preoperative counselling, nutritional optimization, tailored analgesia, minimally invasive surgery, early mobilization, and early feeding- implemented across all phases of care [7].

In elective colorectal surgery, the adoption of ERAS pathways has consistently demonstrated reductions in postoperative complications, earlier return of bowel function, and shorter LOS without increases in mortality or readmission rates [8–10]. On this basis, ERAS is now regarded as the standard of care, as reflected in the most recent ERAS Society guidelines for elective colorectal surgery (2025 update) [11], as well as in the joint clinical practice guidelines from ASCRS and SAGES [12].

However, much of the available evidence on ERAS efficacy derives from heterogeneous colorectal populations dominated by malignant disease. The extent to which these benefits extend to benign colorectal conditions—particularly diverticular disease—remains insufficiently explored [12]. Patients undergoing elective surgery for diverticular disease differ from those with colorectal cancer in several clinically relevant respects, including chronic inflammatory changes, anatomical distortion of the sigmoid colon and mesentery, and a history of recurrent inflammatory episodes or prior interventions. Such factors may influence operative complexity, postoperative pain, gastrointestinal function, and overall recovery, raising important questions about whether ERAS pathways validated primarily in oncologic cohorts are equally effective and safe in purely benign diverticular disease. Notably, evidence from inflammatory bowel disease also supports the applicability of enhanced recovery pathways in benign inflammatory colorectal conditions. A recent systematic review demonstrated that ERAS improves postoperative outcomes and accelerates recovery in IBD patients, despite the heightened inflammatory burden and surgical complexity characteristic of these disorders [13].

Current data specifically evaluating ERAS in this context are limited. Several series of minimally invasive sigmoidectomy performed within ERAS-aligned perioperative frameworks suggest that enhanced recovery principles are feasible and may facilitate earlier recovery, improved pain control, and reduced LOS [14, 15]. However, robust comparative evidence in homogeneous benign diverticular cohorts remains scarce.

Addressing these gaps is essential to refine perioperative pathways in this setting. The present study was therefore designed to evaluate the impact of a standardised ERAS programme on postoperative outcomes in patients undergoing elective colorectal resection for diverticular disease, compared with conventional perioperative care. By restricting the analysis to a purely benign, non-oncologic cohort and employing a clearly defined ERAS versus non-ERAS comparison, this study aims to provide clinically relevant evidence to inform best practice in the perioperative management of diverticular disease.

## Methods

### Study design and setting

This was a retrospective, multicentre cohort study including all consecutive adult patients who underwent elective colorectal resection for left-sided diverticular disease between January 2009 and December 2024 across participating hospitals in Northern Italy. The study was designed and reported in accordance with the STROBE guidelines for observational cohort studies [16]. The study compared perioperative and postoperative outcomes between patients managed within a standardized Enhanced Recovery After Surgery (ERAS) programme (Group A) and those treated with conventional perioperative care (Group B). The ERAS pathway was jointly agreed upon and implemented across participating centres, with uniform core elements and no centre-specific protocol variations.

### Patient selection and eligibility criteria

Eligible patients were adults (≥ 18 years) undergoing elective left-sided colorectal resection for diverticular disease confirmed clinically and radiologically. Emergency cases, right-sided resections, surgeries performed for malignant disease, and procedures combined with other major abdominal operations were excluded. Patients were managed according to the perioperative pathway in use at the time of surgery: a conventional care protocol was employed during the initial study period, followed by the progressive introduction and full implementation of a standardized ERAS programme. Accordingly, patients were allocated to the ERAS or non-ERAS group based on the institutional protocol active at the time of their operation and surgeon adherence to the pathway.

### Perioperative care pathways-ERAS programme (Group A)

The ERAS pathway was based on ERAS Society recommendations for colorectal surgery [12, 17] and included the following components: preoperative counselling and nutritional optimization, avoidance of prolonged fasting; carbohydrate loading when appropriate, multimodal analgesia to minimize opioid use, early oral intake with goal-directed fluid therapy (liquids on POD 0–1; advancement as tolerated), standardized infection preventive measures, minimally invasive surgical approaches when feasible, early mobilization (target within 12 h postoperatively), avoidance of routine nasogastric tubes and drains, standardized criteria for discharge readiness. Compliance with individual ERAS elements was not formally quantified, as adherence was assessed at the pathway level rather than through item-specific auditing.

### Conventional care (Group B)

The non-ERAS cohort received traditional perioperative management, which generally included: variable fasting protocols, non-standardized postoperative analgesia (more frequent opioid use), delayed oral intake, delayed mobilization (commonly > 18–24 h), routine or selective use of abdominal drains, no structured protocol defining postoperative milestones.

### Data collection

Data extraction was performed independently by two reviewers (SL and FB). Any discrepancies or uncertainties were discussed and resolved by consensus with a third senior reviewer (DC). Data were retrieved from institutional electronic medical records and included: demographics and comorbidities, American Society of Anesthesiology (ASA) score, bowel preparation, type of operation and surgical approach, operative time, intraoperative blood loss and conversion rate, need for postoperative intensive care unit (ICU) admission, postoperative pain (VAS score on POD 1), postoperative blood transfusions, time to first flatus and stool, time to oral intake and mobilization, removal of drains, postoperative complications classified according to Clavien–Dindo, need for reoperation, 30-day readmission, length of hospital stay (LOS), 30-day mortality. Disease complexity variables, including the presence of fistulizing diverticulitis and colonic stenosis, were also recorded. Early mobilization was defined as ambulation within the first 24 postoperative hours. Gastrointestinal recovery was defined as the time to first passage of flatus and the time to first bowel movement, recorded in days from the index operation.

### Outcomes

The primary outcome of this study was length of hospital stay (LOS), defined as the number of days from the index operation to hospital discharge. LOS was selected as the primary endpoint because it represents a comprehensive indicator of postoperative recovery, integrating functional recovery, complication burden, and readiness for discharge.

Secondary outcomes included postoperative morbidity, gastrointestinal recovery, postoperative pain, early mobilisation, intensive care unit admission, reoperation, readmission, and 30-day mortality.

Postoperative morbidity was defined as any complication occurring within 30 days of surgery and was classified according to the Clavien–Dindo classification. Overall morbidity included all grades (I–V), while major morbidity was defined as Clavien–Dindo grade III or higher.

Gastrointestinal recovery was assessed using time to first passage of flatus and time to first bowel movement, measured in postoperative days.

Postoperative pain was assessed using the visual analogue scale (VAS). In this retrospective cohort, VAS scores were routinely and systematically recorded on postoperative day 1 as part of standard clinical documentation. No additional standardized serial pain measurements were consistently available beyond POD 1.

Within the ERAS pathway, early mobilisation targeted ambulation within 12 h postoperatively. For analytical consistency across both cohorts, early mobilisation was defined as ambulation within the first 24 postoperative hours. Additionally, time to mobilisation was analysed as a continuous variable (measured in hours from the end of surgery) to provide a more detailed assessment of mobilisation timing.

Hospital readmission was defined as any unplanned hospital admission occurring within 30 days following discharge.

### Statistical analysis

Statistical analyses were performed to compare perioperative and postoperative outcomes between patients managed within the ERAS pathway and those receiving conventional care. The primary analysis compared the length of hospital stay between groups, while secondary analyses evaluated postoperative morbidity, gastrointestinal recovery, postoperative pain, mobilisation, and readmission.

Continuous variables were assessed for distribution using visual inspection and summary statistics, reported as median and interquartile range, and compared using the Mann–Whitney U test given their non-parametric nature. Categorical variables were summarised as frequencies and percentages and compared using the χ^2^ test or Fisher’s exact test when cell counts were < 5. A two-sided p-value < 0.05 was considered indicative of statistical significance. No imputation was performed for missing data, as completeness exceeded the threshold for reliable case-wise analysis. Given the observational study design, no adjustment for multiplicity or modelling of causal associations was undertaken; results were interpreted as comparative rather than predictive. Statistical analyses were performed using Python, employing the SciPy and Pandas libraries, which provided validated implementations of non-parametric and categorical comparison methods.

### Propensity score analysis

A propensity score matching (PSM) procedure was performed as a secondary analysis to account for potential baseline and temporal confounding between ERAS and non-ERAS patients. The propensity score represented the probability of being treated within the ERAS pathway and was estimated using a multivariable logistic regression model including the following pre-treatment variables: age, sex, BMI, ASA class (I–II vs III–IV), hypertension, diabetes mellitus, cardiovascular disease, smoking status, history of recurrent diverticulitis (≥ 2 episodes), previous abdominal surgery, and period of surgery. Patients were matched 1:1 using nearest-neighbour matching without replacement, with a caliper width of 0.2 of the standard deviation of the logit of the propensity score. Covariate balance before and after matching was assessed using standardized mean differences (SMD), with values < 0.1 considered indicative of adequate balance. The same statistical tests used in the primary analysis were applied to the matched cohort. All steps of the PSM procedure were performed in Python using validated implementations of logistic regression and nearest-neighbour matching.

*Ethical approval.*

The study was conducted in accordance with the Declaration of Helsinki and approved by the Institutional Ethics Committee of the participating hospital.

## Results

**Fig. 1:**
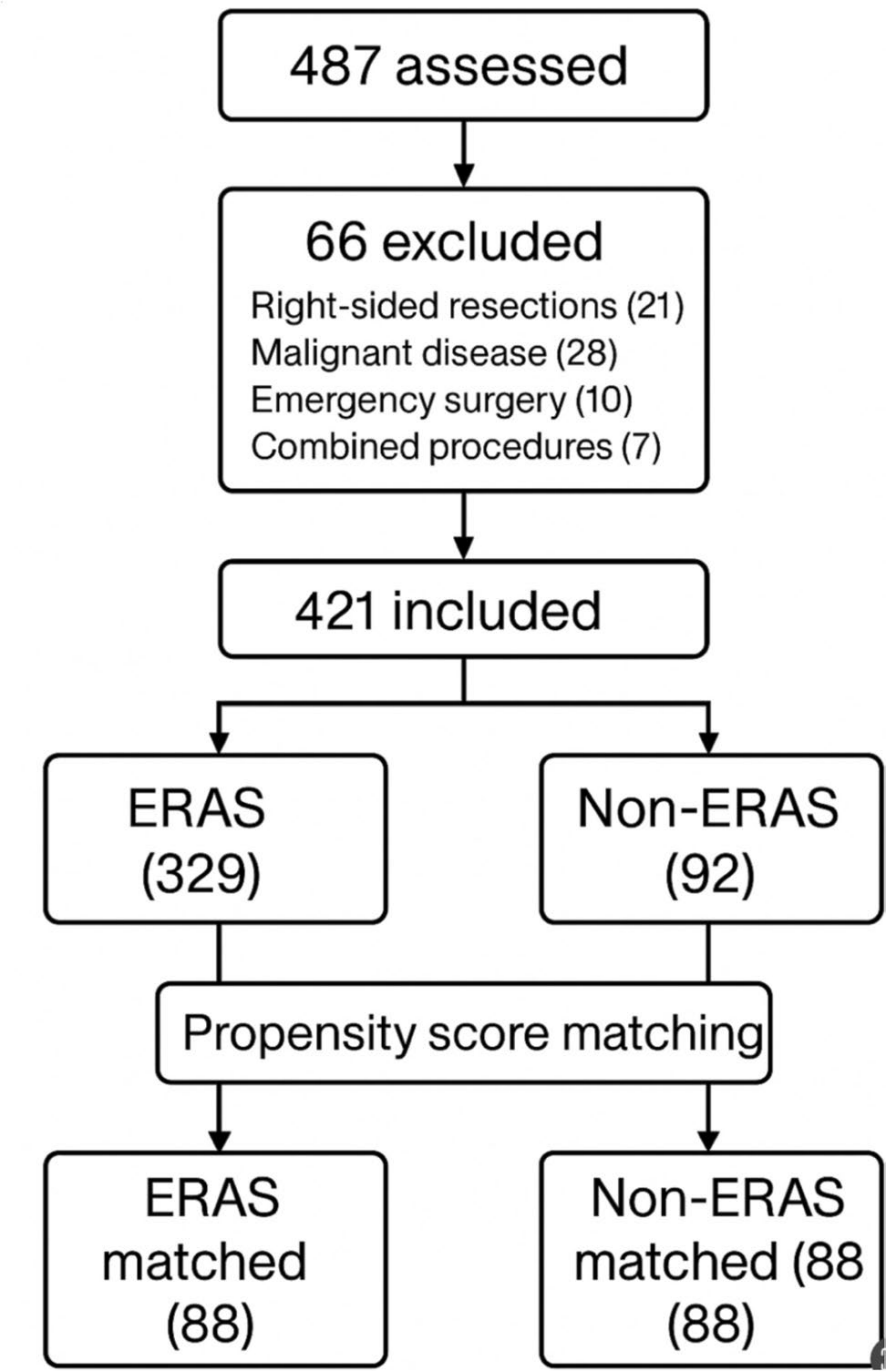
Patient selection and study cohort formation

During the study period, 487 patients undergoing elective colorectal resection were assessed for eligibility. Sixty-six were excluded due to right-sided resections (n = 21), malignant disease (n = 28), emergency surgery (n = 10), or combined major abdominal procedures (n = 7). A total of 421 patients undergoing elective left-sided colorectal resection for diverticular disease were therefore included in the analysis (Fig. 1). Of these, 329 were managed within an ERAS pathway and 92 received conventional perioperative care. To address potential baseline and temporal confounding, a propensity score–matched cohort of 88 patient pairs (n = 176) was subsequently generated, yielding matched ERAS (n = 88) and matched non-ERAS (n = 88) groups for sensitivity analysis. Baseline demographic and intraoperative characteristics were comparable across the two cohorts. (Table 1) The proportion of patients with complicated diverticular disease was similar between groups. Fistulizing diverticulitis was present in 10 patients (3.0%) in the ERAS group and 6 patients (6.5%) in the non-ERAS group. Colonic stenosis was observed in 12 ERAS patients (3.6%) and 7 non-ERAS patients (7.6%), with no clinically meaningful imbalance between cohorts.

**Table 1:** Baseline demographic and intraoperative characteristics of the study population

Characteristic	ERAS Group (n = 329)	Non-ERAS Group (n = 92)	p-value*
**Age, years (mean ± SD)**	63.8 ± 10.7	65.1 ± 11.2	0.28
**Male sex, n (%)**	168 (51.1%)	50 (54.3%)	0.58
**BMI, kg/m**^**2**^** (mean ± SD)**	27.3 ± 3.9	27.7 ± 4.2	0.41
**ASA class, n (%)**			0.47
– I–II	264 (80.2%)	70 (76.1%)	
– III–IV	65 (19.8%)	22 (23.9%)	
**Active smoker, n (%)**	55 (16.7%)	18 (19.6%)	0.49
**Comorbidities, n (%)**			
– Hypertension	147 (44.7%)	41 (44.6%)	0.99
– Diabetes mellitus	42 (12.8%)	14 (15.2%)	0.53
– Cardiovascular disease	36 (10.9%)	13 (14.1%)	0.38
**Recurrent diverticulitis (≥ 2 episodes), n (%)**	198 (60.2%)	55 (59.8%)	0.95
**Previous abdominal surgery, n (%)**	72 (21.9%)	23 (25.0%)	0.52
**Fistulizing diverticulitis**	10 (3.0%)	6 (6.5%)	0.12
**Colonic stenosis**	12 (3.6%)	7 (7.6%)	0.15
**Surgical approach, n (%)**			0.31
– Laparoscopic	292 (88.7%)	78 (84.8%)	
– Robotic	12 (3.6%)	2 (2.2%)	
– Open	25 (7.6%)	12 (13.0%)	
**Operative time, minutes (median [IQR])**	148 [130–170]	152 [135–180]	0.22
**Estimated blood loss, mL (median [IQR])**	80 [40–120]	90 [45–150]	0.19
**Conversion to open surgery, n (%)**	10 (3.0%)	4 (4.3%)	0.49

Postoperative recovery was generally faster and more favorable among patients treated under the ERAS pathway. Pain control on postoperative day 1 was notably improved, with ERAS patients reporting a median VAS score of 2 compared with 4 in the non-ERAS group. A similar trend was observed in gastrointestinal recovery: the time to first passage of flatus occurred at a median of one day in the ERAS cohort versus two days in the standard-care cohort, while the return of bowel movements followed a comparable pattern (median of one day vs two days, respectively). Consistent with ERAS principles, solid diet was introduced earlier in the ERAS group (POD 1), whereas patients receiving conventional care advanced to solid intake at POD 2. Mobilization was significantly earlier among ERAS patients, who achieved ambulation at approximately 12 h, compared with 21 h in those treated with conventional care. Although drain placement was significantly less frequent in the ERAS group (4.25% vs. 100%, p < 0.001), the timing of drain removal was similar between groups, with a median of two postoperative days.

Postoperative morbidity was lower in the ERAS cohort. Overall complications occurred in 6.6% of ERAS patients compared with 14% in the non-ERAS group. Minor complications (Clavien–Dindo I–II) were less frequent in the ERAS arm (1.8% vs 5.4%), and major morbidity (Clavien–Dindo III–IVb) also showed a reduction (4.8% vs 8.6%). Reoperations (Clavien–Dindo IIIb) were required in 3% of ERAS patients and in 4.3% of non-ERAS patients. Postoperative ICU admission occurred in 3.6% of ERAS patients compared with 6.5% in the non-ERAS group. The need for blood transfusion remained low overall and did not differ substantially between cohorts. Mortality at 30 days was rare and occurred only in the conventional-care group (0.3%).

Length of hospital stay was significantly shorter among patients treated within the ERAS programme. The median LOS was 4 days in the ERAS cohort, compared with 6 days in the non-ERAS cohort. Consistently, 30-day readmissions were numerically lower in the ERAS group (1.2%) versus conventional care (2.2%) (Table 2).

**Table 2:** Postoperative outcomes in patients undergoing elective colorectal resection for diverticular disease, according to perioperative pathway (unmatched analysis)

Outcome	ERAS (n = 329)	Non-ERAS (n = 92)	p-value
Pain POD1 (VAS)	2 (1–3)	4 (3–5)	< 0.001
Time to first flatus, days	1 (1–2)	2 (1–2)	< 0.001
Time to first stool, days	1 (1–2)	2 (2–3)	< 0.001
Early mobilisation, hours	12 (10–18)	21 (18–30)	< 0.001
Solid diet (POD)	1	2	< 0.001
Drain placement, n (%)	14 (4.25)	92 (100)	< 0.001
Drain removal, POD	2 (2–3)	2 (2–3)	0.41
Overall morbidity, n (%)	22 (6.6%)	13 (14.1%)	0.02
Minor complications (CD I–II), n (%)	6 (1.8%)	5 (5.4%)	0.06
Major complications (CD III–IV), n (%)	16 (4.8%)	8 (8.6%)	0.19
Reoperations, n (%)	10 (3.0%)	4 (4.3%)	0.49
ICU admission, n (%)	12 (3.6%)	6 (6.5%)	0.21
Blood transfusion, n (%)	4 (1.2%)	2 (2.2%)	0.43
30-day readmission, n (%)	4 (1.2%)	2 (2.2%)	0.48
Length of stay, days	4 (3–6)	6 (5–8)	< 0.001
30-day mortality, n (%)	0 (0%)	1 (1.1%)	0.18

### Propensity score–matched analysis

Propensity score matching generated 88 matched pairs (n = 176 patients), representing the largest feasible 1:1 matched cohort based on the size of the non-ERAS group. After matching, all baseline covariates included in the propensity model demonstrated excellent balance between groups, with standardized mean differences < 0.1, confirming the effectiveness of the matching procedure (Table 3).

**Table 3:** Baseline demographic and clinical characteristics after propensity score matching

Characteristic	ERAS (n = 88)	Non-ERAS (n = 88)	SMD
Age, years (mean ± SD)	64.1 ± 10.5	64.7 ± 10.8	0.06
Male sex, n (%)	46 (52.3%)	45 (51.1%)	0.03
BMI, kg/m^2^ (mean ± SD)	27.5 ± 4.0	27.6 ± 4.1	0.02
ASA III–IV, n (%)	18 (20.5%)	19 (21.6%)	0.03
Hypertension, n (%)	40 (45.5%)	39 (44.3%)	0.02
Diabetes mellitus, n (%)	12 (13.6%)	13 (14.8%)	0.03
Cardiovascular disease, n (%)	10 (11.4%)	11 (12.5%)	0.03
Active smoker, n (%)	16 (18.2%)	15 (17.0%)	0.03
Recurrent diverticulitis ≥ 2, n (%)	52 (59.1%)	51 (58.0%)	0.02
Previous abdominal surgery, n (%)	19 (21.6%)	20 (22.7%)	0.03
Period of surgery	Balanced	Balanced	< 0.05

Within the matched cohort, the direction and magnitude of the differences between ERAS and non-ERAS patients remained fully consistent with the primary analysis. ERAS adherence continued to be associated with faster gastrointestinal recovery (time to first flatus: 1 vs 2 days; time to first stool: 1 vs 2 days; both p < 0.001), lower pain scores on POD 1 (VAS 2 vs 4, p < 0.001), earlier mobilisation (12 h vs 20 h, p < 0.001), and earlier introduction of solid diet (POD 1 vs POD 2, p < 0.001). Length of stay remained significantly shorter in the ERAS group (median 4 vs 6 days, p < 0.001). Postoperative morbidity showed a similar trend to the main analysis, with lower overall complications in ERAS patients (6.8% vs 12.5%, p = 0.21) and no increase in major complications, reoperations, ICU admission, or 30-day readmissions (Table 4). No increase in postoperative complications or adverse outcomes was observed.

**Table 4:** Postoperative outcomes after propensity score matching

Outcome	ERAS (n = 88)	Non-ERAS (n = 88)	p-value
Time to first flatus, days	1 (1–1)	2 (1–2)	< 0.001
Time to first stool, days	1 (1–2)	2 (2–3)	< 0.001
Pain POD1 (VAS)	2 (1–3)	4 (3–5)	< 0.001
Early mobilisation, hours	12 (10–16)	20 (16–28)	< 0.001
Solid diet (POD)	1	2	< 0.001
Overall morbidity, n (%)	6 (6.8%)	11 (12.5%)	0.21
Major morbidity (CD III–IV), n (%)	4 (4.5%)	7 (7.9%)	0.37
Reoperations, n (%)	2 (2.3%)	3 (3.4%)	0.65
ICU admission, n (%)	3 (3.4%)	5 (5.7%)	0.42
30-day readmission, n (%)	1 (1.1%)	2 (2.3%)	0.56
Length of stay, days	4 (4–5)	6 (5–7)	< 0.001

## Discussion

In this large retrospective cohort of patients undergoing elective colorectal resection for left-sided diverticular disease, adherence to an ERAS programme was associated with significantly improved postoperative recovery and lower overall morbidity compared with conventional perioperative care. These findings reinforce the applicability and clinical value of ERAS principles in benign colorectal conditions, a setting in which high-quality evidence has historically been limited.

The accelerated functional recovery observed in the ERAS cohort—reflected by earlier return of flatus and stool, reduced postoperative pain, and earlier ambulation—is consistent with the established physiological rationale of ERAS pathways. Minimisation of perioperative fasting, structured multimodal analgesia, optimisation of fluid therapy, and early mobilisation collectively mitigate the postoperative stress response and the duration of ileus, thereby facilitating earlier restoration of gastrointestinal function. These physiological effects are supported by randomized evidence demonstrating that ERAS protocols significantly reduce postoperative inflammatory response, including IL-6 secretion, in patients undergoing laparoscopic colorectal surgery, thereby attenuating the overall surgical stress burden [18]. Although ERAS protocols were initially validated predominantly in oncologic colorectal populations, our results demonstrate that the same benefits can be extended to patients with diverticular disease, who often present with chronic inflammation, distorted anatomy and a higher baseline risk of delayed recovery. These benefits are in line with prior evidence showing that ERAS protocols can attenuate the surgical stress response and promote accelerated recovery even in elderly patients undergoing elective laparoscopic colorectal surgery [19].

The improvement in postoperative outcomes observed in this study aligns with existing evidence in mixed colorectal cohorts, where ERAS has been shown to reduce complications, shorten LOS, and enhance patient recovery [9, 10]. However, literature specifically addressing benign colorectal disease—particularly diverticular disease requiring elective resection—remains sparse. The few available series of minimally invasive sigmoidectomy within ERAS frameworks have suggested similar advantages, but most studies are limited by heterogeneous indications, small sample sizes, or an emphasis on technical comparisons rather than perioperative pathways [14, 15, 20]. By focusing exclusively on left-sided diverticular disease and incorporating a contemporaneous non-ERAS control group, our findings help fill this knowledge gap and provide dedicated evidence supporting ERAS implementation in this population.

Of note, the reduction in overall morbidity and the absence of increased readmission rates in the ERAS group reinforce the safety of enhanced recovery protocols in benign colorectal surgery. Patients with diverticular disease are frequently older and present with multiple comorbidities; yet, the structured ERAS pathway did not increase postoperative risks and was instead associated with fewer complications, less need for ICU admission, and improved pain control. These results suggest that ERAS may be particularly beneficial in this patient population by promoting physiological stability and early mobilisation, factors known to reduce the incidence of both minor and major complications.

From a health system perspective, the reduction in length of stay observed in the ERAS group may have relevant economic implications. A median saving of two postoperative hospital days can translate into substantial reductions in direct hospitalization costs, particularly in high-volume colorectal units, while also improving bed availability and operating room throughput. Although a formal cost or cost-effectiveness analysis was beyond the scope of this retrospective study, these findings suggest that ERAS implementation may contribute not only to improved clinical outcomes but also to more efficient use of hospital resources. Future prospective studies incorporating structured economic evaluations are warranted to better quantify the cost impact of ERAS pathways in benign colorectal surgery.

Not all perioperative variables differed between groups. For instance, the timing of drain removal and the need for postoperative transfusions were comparable, likely reflecting standardised institutional practices rather than pathway-specific effects. However, these parallels do not detract from the clinical significance of the improvements observed with ERAS in terms of functional recovery and postoperative morbidity, which remain central determinants of the patient’s postoperative course and overall hospital resource utilisation.

This study has several strengths, including its large sample size, the inclusion of a homogeneous benign cohort, and the real-world evaluation of ERAS implementation over an extended time period. The availability of a contemporaneous non-ERAS group provides a meaningful comparator to assess the impact of structured perioperative pathways in diverticular disease. Importantly, these findings were further supported by propensity score–matched analysis, which achieved excellent covariate balance and confirmed consistent improvements in functional recovery metrics, including earlier gastrointestinal recovery, reduced postoperative pain, earlier mobilisation, and shorter length of stay. These results strengthen the internal validity of the study and suggest that ERAS implementation is associated with improved postoperative recovery in this setting.

However, propensity score matching cannot account for unmeasured or incompletely measured confounders. Factors such as surgeon experience, institutional learning curves, and progressive maturation of ERAS implementation may have influenced outcomes and were not fully captured in the model. Furthermore, the long inclusion period introduces potential temporal bias related to secular improvements in surgical techniques, perioperative care, and multidisciplinary management. Although the period of surgery was included in the propensity score model, residual temporal confounding cannot be entirely excluded. Additionally, the reduced sample size following matching may have limited statistical power to detect differences in less frequent outcomes, such as postoperative morbidity. Therefore, while the consistency of findings across unmatched and matched analyses supports the robustness of the observed associations, these results should be interpreted as demonstrating association rather than definitive causation.

Several additional limitations should be acknowledged. The retrospective design carries an inherent risk of selection bias, and adherence to individual ERAS components may have varied over time, particularly during the early phases of implementation. As ERAS was introduced at a defined time point, the study reflects a retrospective before–after design, which is inherently susceptible to temporal bias despite statistical adjustment.

Although the prevalence of complicated diverticular disease—including fistulizing disease and colonic stenosis—was comparable between groups, these variables were not incorporated into the propensity score model and may represent a potential source of residual confounding. Similarly, surgeon-level factors and institutional experience were not formally adjusted for and may have influenced outcomes. Pain assessment represents an additional limitation of the present study. Postoperative pain was evaluated using a single time-point measurement (VAS on POD 1), reflecting the only consistently documented assessment available within the retrospective dataset. While early postoperative pain is clinically relevant—particularly in the context of mobilisation and feeding milestones—it does not fully capture the longitudinal trajectory of postoperative analgesia. Future prospective studies incorporating serial pain assessments would provide a more comprehensive evaluation of analgesic effectiveness within ERAS pathways.

Another important limitation is the lack of structured quantitative assessment of adherence to individual ERAS components. ERAS effectiveness is closely related to compliance with specific protocol elements, rather than representing a purely binary exposure. Although patients were managed within a standardized ERAS pathway, detailed compliance scoring and component-level adherence—including early feeding, mobilisation, and opioid-sparing analgesia—were not systematically recorded. Consequently, it was not possible to evaluate overall ERAS compliance rates or their relationship with postoperative outcomes. Variability in adherence, particularly during early implementation phases, may have influenced outcome magnitude. Additionally, postoperative opioid consumption could not be analysed quantitatively, as it was not consistently recorded throughout the study period. Prospective studies incorporating standardized ERAS compliance assessment are needed to better define the relationship between protocol adherence and clinical benefit.

Finally, patient-reported outcomes and quality-of-life measures were not assessed. These endpoints are particularly relevant in benign colorectal disease, where functional recovery and symptom resolution represent key determinants of treatment success.

Despite these limitations, this study provides clinically relevant real-world evidence supporting the implementation of ERAS pathways in elective colorectal surgery for diverticular disease. ERAS adoption was consistently associated with faster functional recovery and shorter hospital stay, without evidence of increased morbidity or readmission. These findings support the feasibility and clinical value of structured perioperative care pathways in benign colorectal surgery. Future prospective and multicentre studies, ideally incorporating standardized compliance assessment and patient-reported outcomes, are warranted to further refine ERAS implementation and optimize recovery in this patient population.

## Conclusion

In this large, benign cohort, ERAS implementation in elective colorectal resection for diverticular disease proved safe and effective, leading to faster recovery and shorter hospital stay without compromising postoperative outcomes. These findings support the integration of structured enhanced recovery pathways into routine practice for diverticular disease and underscore the need for prospective studies to refine ERAS strategies in benign colorectal surgery.

## Data Availability

The datasets generated and/or analysed during the current study are available from the corresponding author on reasonable request.
